# Reproducibility of Middle Cerebral Artery Stenosis Measurements by DSA: Comparison of the NASCET and WASID Methods

**DOI:** 10.1371/journal.pone.0130991

**Published:** 2015-06-26

**Authors:** Luguang Chen, Qian Zhan, Chao Ma, Qi Liu, Xuefeng Zhang, Xia Tian, Yuanliang Jiang, Yinmei Dong, Shiyue Chen, Jianping Lu

**Affiliations:** Department of Radiology, Changhai Hospital of Shanghai, The Second Military Medical University, No. 168 Changhai Road, Shanghai, 200433, China; University of New Mexico, UNITED STATES

## Abstract

**Purpose:**

To evaluate the intra- and inter-observer variability of the North American Symptomatic Carotid Endarterectomy Trial (NASCET) and Warfarin-Aspirin Symptomatic Intracranial Disease (WASID) criteria for the evaluation of middle cerebral artery (MCA) stenosis using digital subtraction angiography (DSA).

**Materials and Methods:**

DSA images of 114 cases with 131 stenotic MCAs were retrospectively analyzed. Two radiologists and a researcher measured the degree of MCA stenosis independently using both NASCET and WASID methods. To determine intra-observer agreement, all the observers reevaluated the degree of MCA stenosis 4 weeks later. The linear relation and coefficient of variation (CV) between the measurements made by the two methods were assessed by correlation coefficient and multi-factor analysis of variance (ANOVA), respectively. Intra- and inter-observer variability of the two methods was evaluated by intraclass correlation coefficient (ICC), Spearman’s R value, Pearson correlation coefficient and Bland-Altman plots.

**Results:**

Despite the fact that the degree of MCA stenosis measured by NASCET was lower than measured using the WASID method, there was good linear correlation between the measurements made by the two methods (for the mean measurements of the 3 observers, NASCET% = 0.891 × WASID% - 1.89%; ICC, Spearman’s R value and Pearson correlation were 0.874, 0.855, and 0.874, respectively). The CVs of both intra- and inter-observer measurements of MCA stenosis using WASID were significantly lower than that using NASCET confirmed by the multi-factor ANOVA results, which showed only the measurement methods of MCA stenosis had significant effects on the CVs both in intra- and inter-observer measurements (both *P* values < 0.001). Intra-observer measurements showed good or excellent agreement with respect to WASID and NASCET evaluation (ICC, 0.656 to 0.817 and 0.635 to 0.761, respectively). Good agreement for the WASID evaluation (ICC, 0.592 to 0.628) and for the NASCET evaluation (ICC, 0.529 to 0.568) was observed for inter-observer measurements. Bland-Altman plots demonstrated that the WASID method had better reproducibility and intra-observer agreement than NASCET method for evaluating MCA stenosis.

**Conclusion:**

Both NASCET and WASID methods have an acceptable level of agreement; however, the WASID method had better reproducibility for the evaluation of MCA stenosis, and thus the WASID method may serve as a standard for measuring the degree of MCA stenosis.

## Introduction

Intracranial artery atherosclerosis is increasingly being recognized as a major cause of stroke worldwide, and patients with intracranial steno-occlusive disease have an augmented risk of vascular events [1]. Intracranial arterial stenosis (IAS) corresponds to luminal narrowing of large intracranial arteries [2]. Primary atherosclerosis is the main cause of IAS, although sometimes embolic events can result in severe stenosis [2]. Atherosclerotic IAS usually occurs in the middle cerebral artery (MCA), which is the principle intracranial artery perfusing the cerebral hemispheres [3]. Moreover, patients with symptomatic MCA stenosis have a higher prevalence (12.5%) of stroke than those with asymptomatic MCA disease (2.85%) [4].

Accurate measurement of the degree of stenosis is important to guide treatment decisions in the clinic [5], and several studies have suggested that patients with more than 70% stenosis may benefit in the long-term from artery stenting, while another study showed an increased risk of ischemic recurrence with significant stenosis [6,7]. Several imaging modalities, including digital subtraction angiography (DSA), computed tomography angiography (CTA), and magnetic resonance angiography (MRA) are used to assess intracranial atherosclerosis. However, the standard method for the evaluation of intracranial atherosclerosis is still DSA. North American Symptomatic Carotid Endarterectomy Trial (NASCET) and Warfarin-Aspirin Symptomatic Intracranial Disease (WASID) are the most commonly used methods to evaluate vascular stenosis. Both methods determine the degree of stenosis by taking the diameter of the residual lumen at the site of maximal luminal narrowing, and the evaluation of stenosis is based on the formula: % stenosis = [1 −(D_stenosis_/D_normal_)] × 100 [1,8]. According to this equation, for the NASCET and WASID methods, the normal segment is ideally measured at a site distal and proximal to the stenotic lesion, respectively [9]. Although designed for carotid stenosis, the NASCET method has been widely employed to measure intracranial stenosis [10].

Although measurements of MCA stenosis using the same angiogram differ according to the method used, a mathematical relationship between the two methods can also be evaluated and it is possible to convert a percent stenosis made by one method to another based on the fact that after analyzing the stenosis degree measurements of a large series of patients [11]. Nevertheless, using two different methods may lead to confusion in clinical practice and, in addition, it prevents generalization of research results. Uniformity in reporting treatment results and consistency in patient management necessitates selection of one method as the standard method in measuring degree of MCA stenosis [11]. During this process, reproducibility is an important factor that should be taken into account.

Therefore, the aim of the present study was to evaluate intra- and inter-observer variability of NASCET and WASID criteria for the evaluation of MCA stenosis using DSA and to compare measurements made by the two methods.

## Materials and Methods

### Subjects

This retrospective study was approved by our Institutional Review Board, Shanghai Changhai Hospital Ethics Committee. Signed written informed consent was waived from all participants. A computerized search of the DSA imaging database and medical records from May 2011 through March 2014 at our institution yielded a list of 200 patients who underwent DSA imaging for suspected intracranial atherosclerotic disease using the aforementioned standard clinical protocol. The inclusion criteria for the present study were: ischemic stroke or transient ischemic attack in the target MCA territory within the past 30 days; and a stenosed vessel at the M1 segment of the MCA on DSA images. We excluded patients using the following criteria: 1) poor image quality for interpretation; 2) nonatherosclerotic vasculopathy, such as dissection or Moyamoya disease; and 3) normal or occluded arteries at the M1 segment of the MCA. In the final analysis, 114 subjects with 131 stenosed MCAs were enrolled in this study.

### Digital subtraction angiography

DSA was carried out using a Siemens angiographic unit (Siemens Medical Solution, Erlangen, Germany) with a protocol involving femoral puncture and selective injection of Ultravist contrast agent (Bayer Healthcare, Erlangen, Germany) into the MCA vessels. Images were acquired using a 1024 × 1024 matrix, a 220 × 220 mm^2^ field of view, and a pixel size of 0.21 × 0.21 mm^2^ at 5 mL/s until the late venous phase. Three projections (anteroposterior, oblique, and lateral views) were acquired in all cases.

### Image analysis

**Fig 1:**
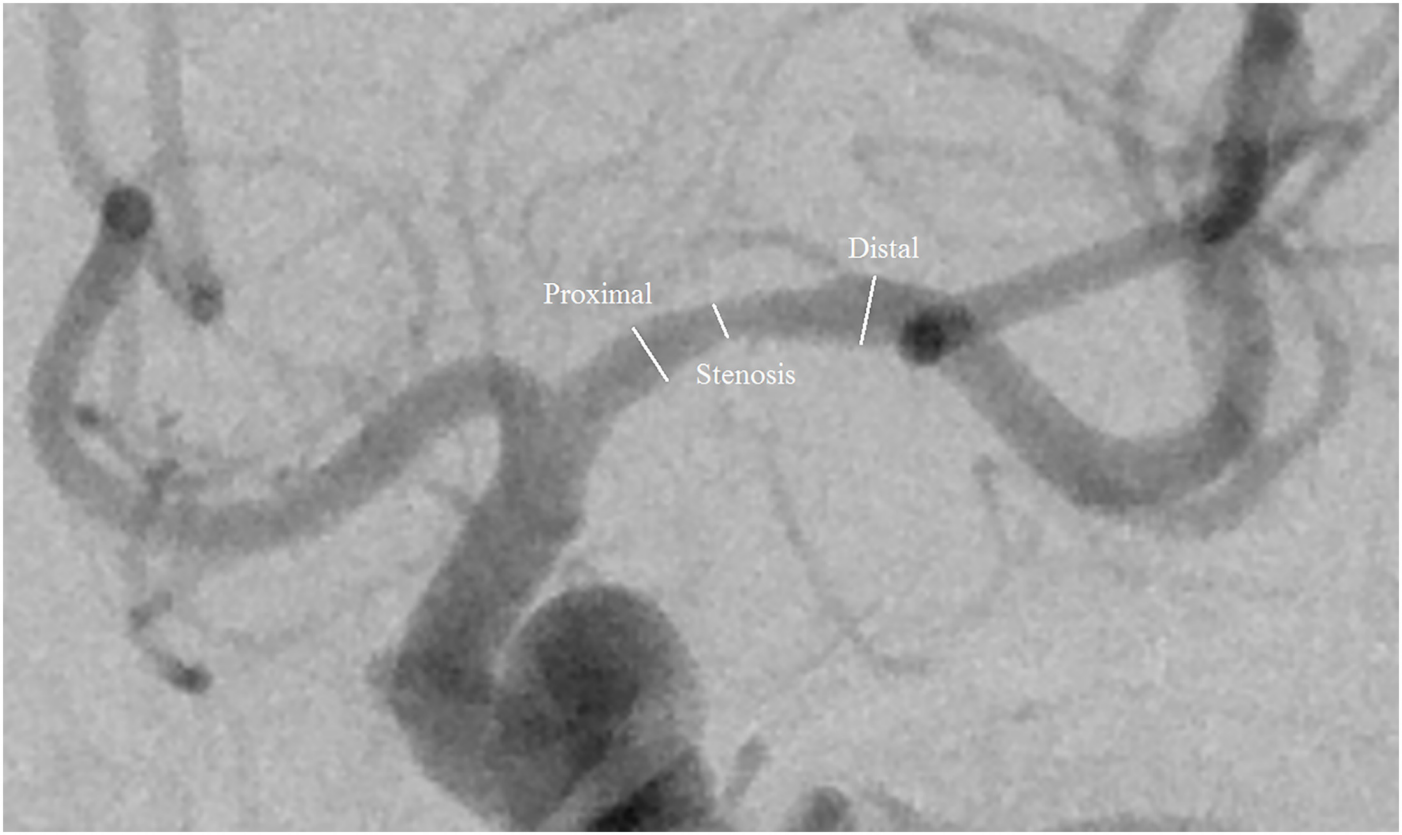
Digital subtraction angiography image showing the points where measurements were taken. The NASCET method uses the distal segment as a comparator to the stenotic region. The WASID method divides the stenotic segment measurement by the proximal normal segment.

All measurements of luminal stenosis were performed by three independent observers: two radiologists (referred to as Observer 1 and 2, respectively) with 5 years of experience in neuroradiology, and one researcher (referred to as Observer 3) with 4 years of experience in neuroradiology, who were blinded to the clinical information of each patient. An electronic ruler from industry-standard Digital Imaging and Communications in Medicine reading software (General Electric Advantage Work-station, GE Healthcare, Milwaukee, WI, USA) configured on a physician workstation with a technical high resolution screen (Jusha Healthcare, Nanjing, People’s Republic of China) was used to measure vessel diameters on the anteroposterior view of DSA images. The images were zoomed to 250%, and window width and level were adjusted to optimize vessel contour. The degree of stenosis or the ratio between the residual lumen at the stenosis and the normal lumen without stenosis was determined using both NASCET and WASID criteria (Fig 1). To evaluate intra-observer variability, three observers measured MCA stenosis twice during two different sessions that were separated by at least 4-week interval to avoid any recall bias. All of the observers were blinded to theirs and each other’s results.

### Statistical analysis

Statistical analyses were performed using the SPSS software for windows (version 16.0, SPSS Inc., Chicago, IL, USA). Quantitative data are described as means ± standard deviation and qualitative data are expressed as percentages. The relationship between measurements of the percentage of MCA stenosis made using NASCET and WASID methods was assessed by correlation coefficient and the results are displayed as scatterplots with four regression lines (the horizontal and vertical axes indicate the average degree of stenosis measured using WASID and NASCET methods, respectively). All values of the measurements are expressed as the mean ± SEM (Standard Error of Mean).

The coefficients of variation (CVs) of intra- and inter-observer measurements were used to evaluate the variability between the two methods. The CV was determined by the standard deviation (SD) of the two matched measurements of each method divided the mean of the two matched measurements of each method (CV = SD/mean × 100%). Multi-factor analysis of variance (ANOVA) (two factors in the present study, methods and observers) was used to assess the variability of CVs of intra- and inter-observer measurements between the two methods. The overall CVs and percentage difference of the MCA stenosis measurements made by the two methods were also calculated and compared, respectively.

Intraclass correlation coefficient (ICC) and Bland-Altman plots were used to assess intra- and inter-observer variability of both the NASCET and WASID methods. The ICC is the proportion of the total variance caused by variation between serial measurements or single measurements by different observers. Values were graded according to the method proposed by Shout and Fleiss [12]: < 0.4, poor agreement; 0.4–0.75, good agreement; > 0.75, excellent agreement. For the Bland-Altman analysis [13], differences in the two measurements were plotted against the mean of the two measurements to assess intra- and inter-observer agreement for each method. Therefore, bias and 95% confidence intervals of the mean difference (limits of agreement) were evaluated across the mean stenosis, and the less agreement, the wider the dispersion of the scatterplot at a given mean measurement. Spearman and Pearson correlation tests were also performed to assess relationships between measurements. A *P*-value < 0.05 was regarded as statistically significant.

## Results

A total of 131 MCA M1 segments of 114 cases (75 males [65.8%], 39 females [34.2%], mean age: 56.8 ± 11.2 years) were retrospectively analyzed. Various degrees of stenosis were also detected in these segments. In 53 patients (46.5%), the stenosis was on the left M1 segment of the MCA, while in 44 patients (38.6%) it was on the right M1 segment of the MCA and in 17 patients (14.9%) it was bilateral.

### Correlation between NASCET and WASID

**Fig 2:**
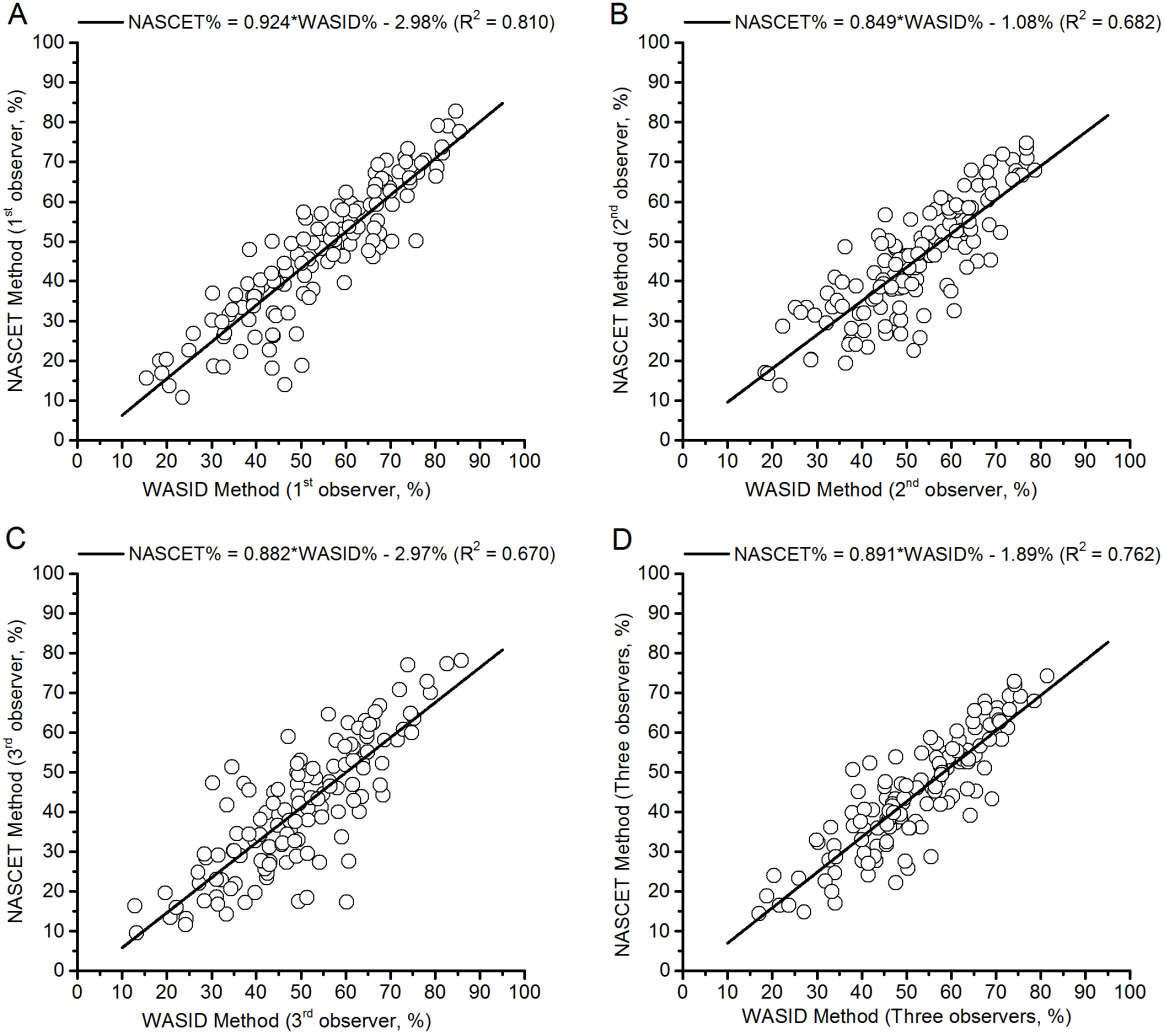
Scatterplots of NASCET and WASID measurements. (A) The measurements of Observer 1. (B) The measurements of Observer 2. (C) The measurements of Observer 3. (D) The mean measurements of all 3 observers.

The results of linear correlation between the NASCET and WASID methods are provided in Table 1. The mean MCA stenosis measurements of each observer and all the 3 observers for NASCET are ranging from 40.9% to 47.3%, and for WASID are ranging from 49.8% to 54.4%. The ICCs, Spearman’s R values and Pearson correlation coefficients of the measurements showed excellent agreement and high correlation between the two methods. The scatterplots showing the NASCET and WASID measurements are shown in Fig 2, where there are four evident linear regression lines between the two criteria measurements. Taking the mean measurements of the 3 observers as an example, the following equation can be deduced from the linear regression line (Fig 2D), and the *R* ^2^ value was 0.762: NASCET% = 0.891 × WASID%– 1.89%; i.e., 70.0% NASCET stenosis is equal to 80.7% WASID stenosis.

**Table 1:** Linear correlation between NASCET and WASID methods in each observer and all 3 observers.

Observer	Mean±SEM (% stenosis)				
	NASCET	WASID	ICC (95%CI)	Spearman’s R value*	Pearson correlation coefficient*
**1^st^**	47.3±1.5	54.4±1.4	0.901 (0.863–0.929)	0.904	0.901
**2^nd^**	45.0±1.2	51.7±1.2	0.828 (0.765–0.875)	0.806	0.828
**3^rd^**	40.9±1.4	49.8±1.3	0.817 (0.751–0.867)	0.795	0.820
**All**	44.4±1.2	52.0±1.2	0.874 (0.826–0.909)	0.855	0.874

SEM, standard error of Mean; NASCET, north American symptomatic carotid endarterectomy trial; WASID, warfarin-aspirin symptomatic intracranial disease; ICC, intraclass correlation coefficient; CI, confidence interval;

* *P* < 0.01.

### Intra-observer agreement

**Fig 3:**
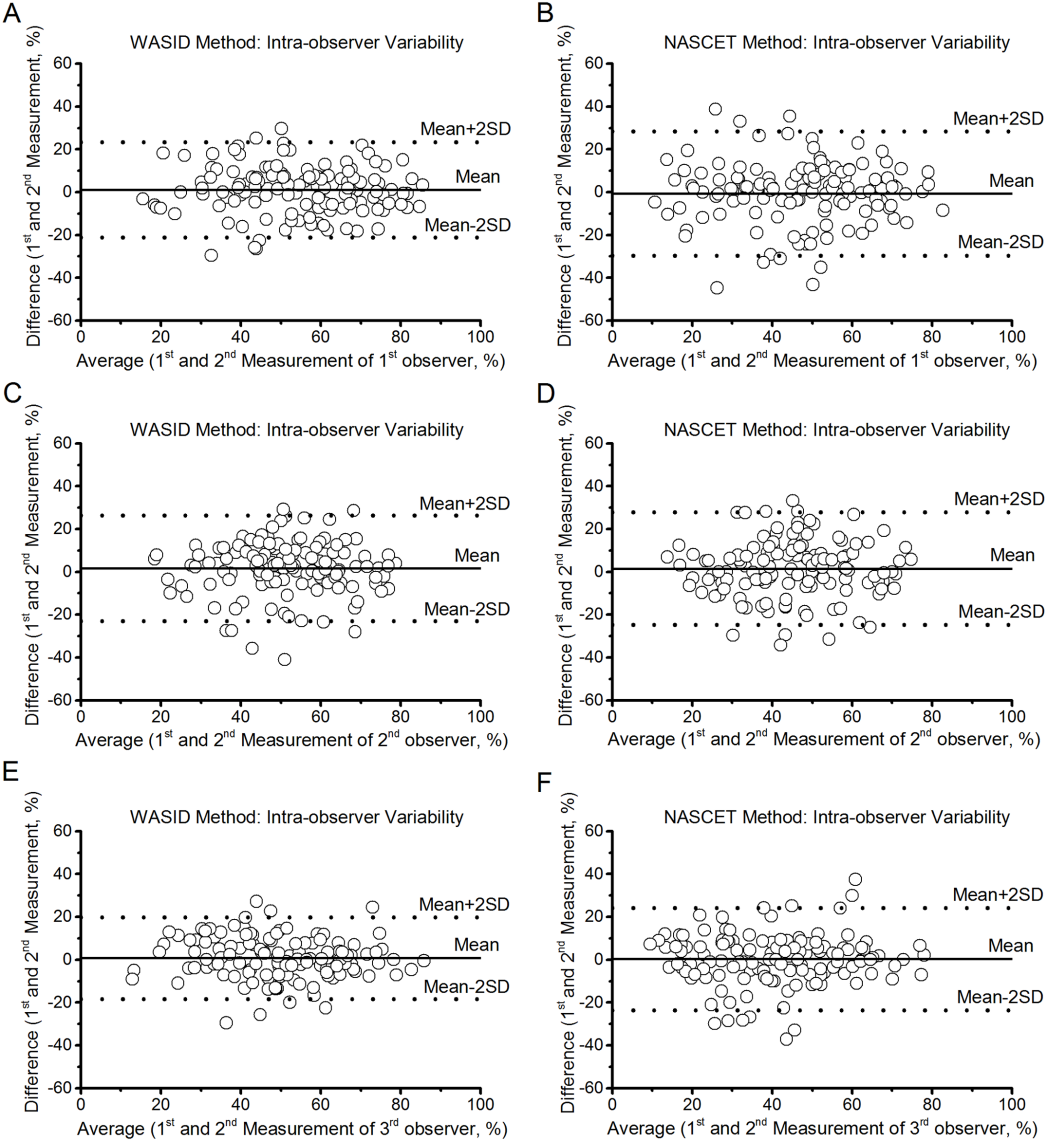
Bland-Altman plots of intra-observer reproducibility of the NASCET and WASID methods. (A, C and E) WASID method for Observer 1, 2 and 3, respectively. (B, D and F) NASCET method for Observer 1, 2 and 3, respectively.

Statistical results of intra-observer reproducibility are presented in Table 2. Intra-observer measurements showed good or excellent agreement both for the NASCET evaluation and the WASID evaluation using ICC (0.635 to 0.761 and 0.656 to 0.817, respectively, *P* < 0.01). Spearman’s R value and Pearson correlation coefficient also suggested a high correlation in intra-observer measurements. The bias and limits of agreement of intra-observer agreement for the measurements of MCA stenosis by using NASCET and WASID methods by employing Bland-Altman plots (Fig 3) were provided in Table 3 (Intra-observer variability). Compared with NASCET, WASID has lower bias and limits of agreement (Table 3) and the narrower the dispersion of the scatterplot at a given measurement (Fig 3). In addition, the WASID method showed better reproducibility than the NASCET method for evaluating of MCA stenosis with a higher ICC (0.791 versus 0.684 for 1^st^ observer, 0.656 versus 0.635 for 2^nd^ observer and 0.817 versus 0.761 for 3^rd^ observer), Spearman’s R value (0.792 versus 0.681 for 1^st^ observer, 0.649 versus 0.624 for 2^nd^ observer and 0.814 versus 0.756 for 3^rd^ observer), and Pearson correlation coefficient (0.791 versus 0.684 for 1^st^ observer, 0.657 versus 0.636 for 2^nd^ observer and 0.818 versus 0.762 for 3^rd^ observer), respectively. Moreover, multi-factor ANOVA results showed the CVs of intra-observer measurements using WASID method were significantly lower than that using NASCET (Table 4) in all of the 3 observers (*P* < 0.001). Observer had no significant effects on the CVs of MCA stenosis in intra-observer measurements (*P* = 0.406).

**Table 2:** Mean±SEM, ICC, Spearman’s R value, and Pearson correlation coefficient statistics of intra-observer agreement.

**Observer**		**Mean±SEM (% stenosis)**				
	**Method**	**The First Measurement**	**The Second Measurement**	**ICC (95%CI)**	**Spearman’s R value** *	**Pearson correlation coefficient** *
**1^st^**	**NASCET**	47.0±1.6	47.7±1.6	0.684 (0.581–0.766)	0.681	0.684
	**WASID**	55.0±1.5	53.9±1.5	0.791 (0.717–0.847)	0.792	0.791
**2^nd^**	**NASCET**	45.7±1.3	44.2±1.3	0.635 (0.521–0.727)	0.624	0.636
	**WASID**	52.5±1.3	50.8±1.3	0.656 (0.547–0.744)	0.649	0.657
**3^rd^**	**NASCET**	41.0±1.5	40.8±1.5	0.761 (0.679–0.825)	0.756	0.762
	**WASID**	50.1±1.3	49.4±1.4	0.817 (0.750–0.867)	0.814	0.818

SEM, standard error of Mean; NASCET, north American symptomatic carotid endarterectomy trial; WASID, warfarin-aspirin symptomatic intracranial disease; ICC, intraclass correlation coefficient; CI, confidence interval;

* *P* < 0.01.

**Table 3:** The mean absolute difference and 95% confidence intervals of the mean difference of intra- and inter-observer variabilities in the evaluation of MCA stenosis.

Method	Intra-observer variability (% stenosis)			Inter-observer variability (% stenosis)		
	Observer 1^st^	Observer 2^nd^	Observer 3^rd^	Observers 1^st^&2^nd^	Observers 1^st^&3^rd^	Observers 2^nd^&3^rd^
**NASCET**	10.6 (-3.2–1.8)	10.2 (-0.7–3.8)	8.8 (-1.8–2.3)	12.7 (-1.6–4.1)	12.6 (3.1–8.8)	12.3 (2.0–7.3)
**WASID**	8.8 (-0.8–3.0)	9.4 (-0.5–3.7)	7.3 (-0.9–2.4)	10.8 (0.0–5.0)	11.5 (2.4–7.3)	10.4 (0.0–4.6)

NASCET, north American symptomatic carotid endarterectomy trial; WASID, warfarin-aspirin symptomatic intracranial disease.

**Table 4:** The mean CVs of intra- and inter-observer of the MCA stenosis measurements between the two methods.

Method	Intra-observer variability (CV %)			Inter-observer variability (CV %)		
	Observer 1^st^	Observer 2^nd^	Observer 3^rd^	Observers 1^st^&2^nd^	Observers 1^st^&3^rd^	Observers 2^nd^&3^rd^
**NASCET**	17.1	17.5	16.9	15.1	18.2	16.8
**WASID**	13.1	14.0	11.3	11.9	13.1	11.3

NASCET, north American symptomatic carotid endarterectomy trial; WASID, warfarin-aspirin symptomatic intracranial disease. the CVs of both intra- and inter-observer measurements of MCA stenosis using WASID were significantly lower than that using NASCET confirmed by the multi-factor ANOVA results, which showed only the measurement methods of MCA stenosis had significant effects on the CVs both in intra- and inter-observer measurements (both *P* values < 0.001).

### Inter-observer agreement

**Fig 4:**
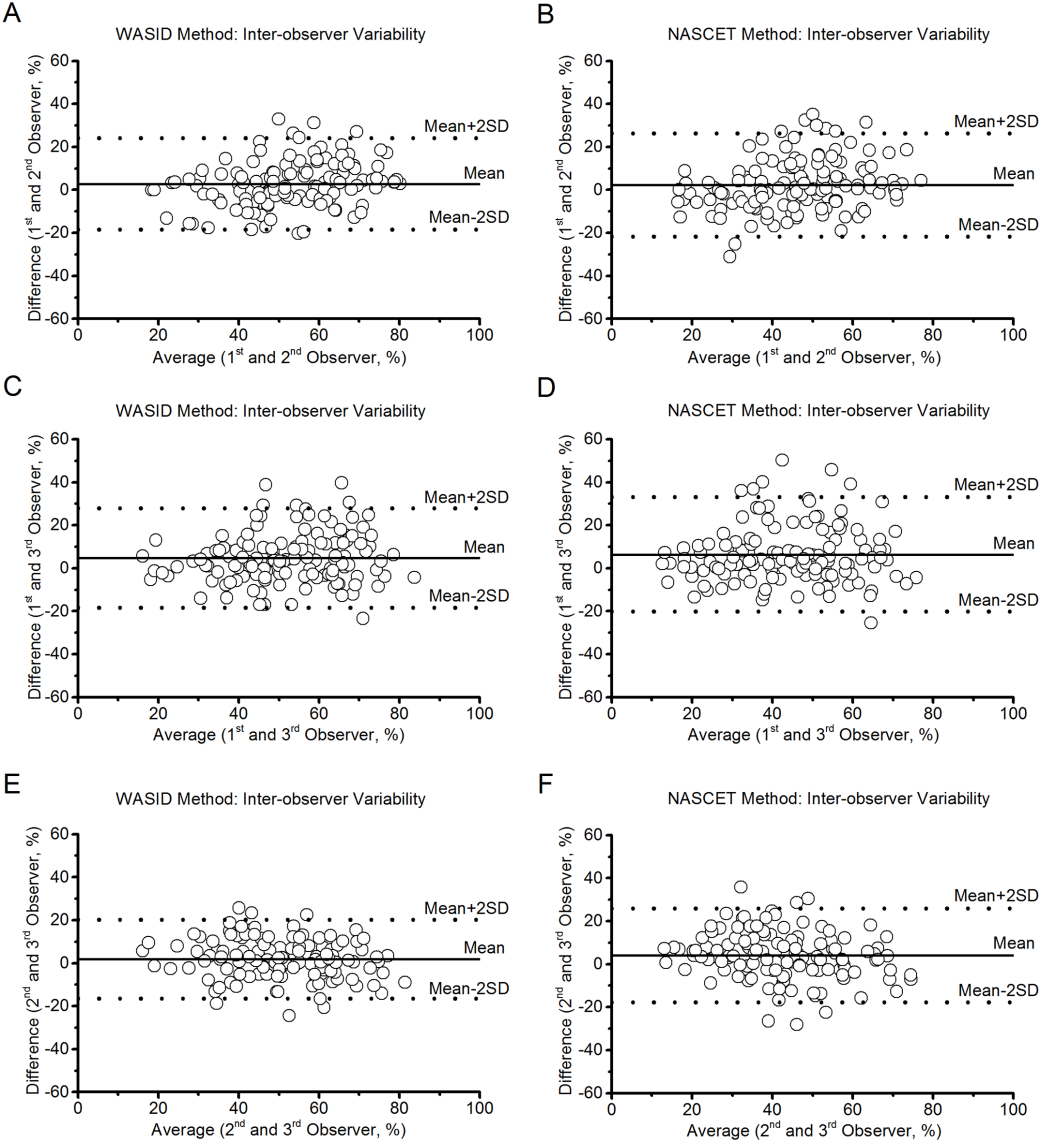
Bland-Altman plots of inter-observer reproducibility of the NASCET and WASID methods. (A, C and E) WASID method for Observers 1&2, 1&3 and 2&3, respectively. (B, D and F) NASCET method for Observers 1&2, 1&3 and 2&3, respectively.

Statistical results of inter-observer reproducibility are presented in Table 5. Inter-observer measurements showed good agreement for both the WASID evaluation and good agreement for the NASCET evaluation using ICC (0.592 to 0.628 and 0.529 to 0.568, respectively, *P* < 0.01). Spearman’s R value and Pearson correlation coefficient also suggested good correlation for inter-observer measurements. The bias and limits of agreement for the measurements of MCA stenosis of inter-observer agreement by employing Bland-Altman plots (Fig 4) for the NASCET and WASID methods were provided in Table 3 (Inter-observer variability), respectively. As with the intra-observer measurements, the WASID method also has lower bias and limits of agreement (Table 3) and the narrower the dispersion of the scatterplot at a given measurement (Fig 4) and had better reproducibility than the NASCET method for evaluating MCA stenosis, as illustrated by higher ICC (0.592 versus 0.529 for the 1^st^ and 2^nd^ observers, 0.628 versus 0.568 for 1^st^ and 3^rd^ observers, and 0.615 versus 0.562 for 2^nd^ and 3^rd^ observers), Spearman’s R value (0.589 versus 0.517 for the 1^st^ and 2^nd^ observers, 0.618 versus 0.560 for 1^st^ and 3^rd^ observers, and 0.605 versus 0.563 for 2^nd^ and 3^rd^ observers), and Pearson correlation coefficient (0.597 versus 0.536 for the 1^st^ and 2^nd^ observers, 0.632 versus 0.569 for 1^st^ and 3^rd^ observers, and 0.615 versus 0.566 for 2^nd^ and 3^rd^ observers), respectively. Moreover, multi-factor ANOVA results showed the CVs of inter-observer measurements using WASID method were significantly lower than that using NASCET (Table 4) in all of the 3 observers (*P* < 0.001). Observer had no significant effects on the CVs of MCA stenosis in inter-observer measurements (*P* = 0.148).

**Table 5:** Mean±SEM, ICC, Spearman’s R value, and Pearson correlation coefficient statistics of inter-observer agreement.

Observers		Mean±SEM (% stenosis)				
	Method	The Former Observer	The Latter Observer	ICC (95%CI)	Spearman’s R value*	Pearson correlation coefficient*
**1^st^ vs. 2^nd^**	**NASCET**	47.0±1.6	45.7±1.3	0.529 (0.394–0.642)	0.517	0.536
	**WASID**	55.0±1.5	52.5±1.3	0.592 (0.468–0.693)	0.589	0.597
**1^st^ vs. 3^rd^**	**NASCET**	47.0±1.6	41.0±1.5	0.568 (0.440–0.673)	0.560	0.569
	**WASID**	55.0±1.5	50.1±1.3	0.628 (0.513–0.722)	0.618	0.632
**2^nd^ vs. 3^rd^**	**NASCET**	45.7±1.3	41.0±1.5	0.562 (0.432–0.669)	0.563	0.566
	**WASID**	52.5±1.3	50.1±1.3	0.615 (0.496–0.711)	0.605	0.615

SEM, standard error of Mean; NASCET, north American symptomatic carotid endarterectomy trial; WASID, warfarin-aspirin symptomatic intracranial disease; ICC, intraclass correlation coefficient; CI, confidence interval;

* *P* < 0.01.

The results showed that the degree of MCA stenosis measured with WASID had lower overall CV than that with NASCET (CVs were 12.5% and 16.9% for WASID and NASCET, respectively), and the percentage difference was 36.0%.

## Discussion

The present study is the first to evaluate the reproducibility of NASCET and WASID techniques for measuring MCA stenosis degree using DSA. Correct quantification of stenosis degree is fundamental in planning the correct therapeutic approach and, because of the confusion generated by the use of different methods, comparative analyses may be important [14]. It is essential to emphasize that the patient population in our study is considerably larger than in previous studies [5, 11]. On the whole, our data indicated good reproducibility of all measurements.

As the NASCET and WASID methods adopt different reference sites for determining normal vessel diameter, each method provides a different degree of stenosis for the same lesion based on the same angiogram [8, 15]. Therefore, referring to the percentage of MCA stenosis without regard for the measurement method is misleading and it may lead to confusion in clinical practice. In our study, however, measurements of MCA stenosis by employing NASCET and WASID methods were highly correlated with Spearman’s R values and Pearson correlation coefficients (Table 1). This correlation was consistent with a previous study from our institute [16]. We also deduced equations based on the scatterplots of NASCET and WASID measurements (Fig 2), which may be used to express the percentage of MCA stenosis measured using one method as a function of the corresponding value measured using another method. However, conversion between the two means of MCA stenosis degree measurements is only possible after analyzing a large series of patients and deriving an equation based on these patients. To our knowledge, no study has previously reported such equations. Moreover, several studies have suggested a linear relationship between the NASCET and WASID methods for the evaluation of carotid artery stenosis [15, 17]. It is worth noting that the degree of stenosis measured with the NASCET method was lower than measured by the WASID method, and the main reason for this is likely the reference points used to determine normal artery diameter by different methods. More specifically, the diameter of the proximal site of MCA is larger than the distal site in accordance with the natural morphology of the vessel [18]; in another words, the diameter of the reference lumen at the proximal site is larger than at the distal site. On the other hand, sometimes the overlapped vessels at the distal site of MCA results in difficulties discriminating the boundaries of the vessels and increases error when evaluating the degree of MCA stenosis [19].

Even though it is possible to convert the degree of stenosis calculated by one method to another method, the best solution is likely to use one method as the current standard [11]. An important factor in choosing a standard method is the level of reproducibility. In our study, with respect to both intra- and inter-observer agreement, we observed good results using NASCET and WASID methods. Moreover, the ICC values of intra- and inter-observer agreement indicated good or excellent and good agreement, respectively (Tables 2 and 5). In addition, the Spearman’s R values and Pearson correlation coefficients of the measurements obtained from different observers using different methods demonstrated to be of high relevance. Yet, the ICCs, Spearman’s R values, and Pearson correlation coefficients of the measurements obtained using WASID method were higher than using NASCET; i.e., Measurements of MCA stenosis using the WASID method were more reproducible than the NASCET method. Moreover, we employed the Bland-Altman plots to evaluate intra- and inter-observer agreement, and the diagrams showed a relatively wide interval of agreement compared with the average (Figs 3 and 4). This suggests possible discrepancies between the two methods and among the 3 observers. However, intra- and inter-observer variability was still acceptable. Furthermore, the CVs of both intra- and inter-observer measurements of MCA stenosis using WASID were significantly lower than that using NASCET confirmed by the multi-factor ANOVA results, which showed only the measurement methods of MCA stenosis had significant effects on the CVs both in intra- and inter-observer measurements (both *P* values < 0.001). C1R2In addition, our analysis also showed that the overall CVs of the measurements made by NASCET and WASID methods were 16.9% and 12.5%, respectively, and WASID has lower CV than NASCET. The variability of measurements of MCA stenosis employing the WASID method has an overall percentage difference of 36.0 than the NASCET method. According to the results of the present study, we found that the WASID has better reproducibility than the NASCET for the evaluation of MCA stenosis, and reproducibility of intra-observer measurements were superior to inter-observer measurements. Several previous studies have also shown the WASID method has better agreement for intra- and inter-observer measurements of carotid artery stenosis [17, 20].

To our knowledge, patients with a greater degree of stenosis of intracranial arteries are more likely to undergo pronounced clinical events and these patients are also more likely to experience symptom recurrence within the same territory as the stenosed arteries [21, 22]. Therefore, accurate measurement of the degree of stenosis and detection of intracranial steno-occlusive disease are vitally important, especially when planning stent surgeries for patients. In the present study, we found that the WASID method is more reproducible, and our results consistent with Samuels et al. [8] in which the WASID method was deemed a better candidate for the worldwide standard for measuring intracranial stenosis on angiograms, as well as for measuring stenosis using non-invasive techniques.

## Conclusions

In conclusion, the present study showed a linear relationship between the NASCET and WASID methods for measuring MCA stenosis. Both NASCET and WASID methods have an acceptable level of agreement; however, the WASID method provided higher values and offered better reproducibility, therefore, it is better suited to serve as the standard for measuring the degree of MCA stenosis in future studies.
